# Ethnic Disparities in Diabetes Management and Pay-for-Performance in the UK: The Wandsworth Prospective Diabetes Study

**DOI:** 10.1371/journal.pmed.0040191

**Published:** 2007-06-12

**Authors:** Christopher Millett, Jeremy Gray, Sonia Saxena, Gopalakrishnan Netuveli, Kamlesh Khunti, Azeem Majeed

**Affiliations:** 1 Wandsworth Primary Care Research Centre, Wandsworth Primary Care Trust, London, United Kingdom; 2 Department of Primary Care and Social Medicine, Imperial College, London, United Kingdom; 3 Department of Health Sciences, Leicester General Hospital, University of Leicester, Leicester, United Kingdom; University of Newcastle, United Kingdom

## Abstract

**Background:**

Pay-for-performance rewards health-care providers by paying them more if they succeed in meeting performance targets. A new contract for general practitioners in the United Kingdom represents the most radical shift towards pay-for-performance seen in any health-care system. The contract provides an important opportunity to address disparities in chronic disease management between ethnic and socioeconomic groups. We examined disparities in management of people with diabetes and intermediate clinical outcomes within a multiethnic population in primary care before and after the introduction of the new contract in April 2004.

**Methods and Findings:**

We conducted a population-based longitudinal survey, using electronic general practice records, in an ethnically diverse part of southwest London. Outcome measures were prescribing levels and achievement of national treatment targets (HbA_1c_ ≤ 7.0%; blood pressure [BP] < 140/80 mm Hg; total cholesterol ≤ 5 mmol/l or 193 mg/dl). The proportion of patients reaching treatment targets for HbA_1c_, BP, and total cholesterol increased significantly after the implementation of the new contract. The extents of these increases were broadly uniform across ethnic groups, with the exception of the black Caribbean patient group, which had a significantly lower improvement in HbA_1c_ (adjusted odds ratio [AOR] 0.75, 95% confidence interval [CI] 0.57–0.97) and BP control (AOR 0.65, 95% CI 0.53–0.81) relative to the white British patient group. Variations in prescribing and achievement of treatment targets between ethnic groups present in 2003 were not attenuated in 2005.

**Conclusions:**

Pay-for-performance incentives have not addressed disparities in the management and control of diabetes between ethnic groups. Quality improvement initiatives must place greater emphasis on minority communities to avoid continued disparities in mortality from cardiovascular disease and the other major complications of diabetes.

## Abstract 2

Based on a population-based longitudinal survey, Christopher Millett and colleagues concluded that pay-for-performance incentives for UK general practitioners had not addressed disparities in the management and control of diabetes between ethnic groups.

## Editors' Summary

**Background.:**

When used in health care, the term “pay-for-performance” means rewarding health-care providers by paying them more if they succeed in meeting performance targets set by the government and other commissioners of health care. It is an approach to health service management that is becoming common, particularly in the US and the UK. For example, the UK's general practitioners (family doctors) agreed with the government in 2004 that they would receive increases to their income that would depend on how well they were judged to be performing according to 146 quality indicators that cover clinical care for ten chronic diseases, as well as “organization of care,” and “patient experience.” One of the chronic diseases is diabetes, a condition that has reached epidemic proportions in the UK, as it has also in many other countries.

Ethnic minorities often suffer more from health problems than the majority population of the country they live in. They are also likely to be served less well by the health services. Diabetes is a case in point; in many countries—including the US and UK—the condition is much more common in minority groups. In addition, their diabetes is usually less well “managed”—i.e., it becomes more severe more rapidly and there are more complications. In the UK, the government recognizes the need to ensure that its health policies are applied to all sectors of the population, including minority ethnic communities. Nevertheless, the advances that have been made in the management of diabetes have not benefited the UK's ethnic minorities to the same extent as they have the majority population. It is hoped that the use of pay-for-performance management by the UK National Health Service will lead to more efficient delivery of health care, and that one consequence will be that different communities will be more equally served.

**Why Was This Study Done?:**

The researchers wanted to find out whether the introduction of pay-for-performance management in general medical practice in the UK was leading to a reduction in the gap in the quality of care provided to people with diabetes who belonged to ethnic minorities and other people with diabetes.

**What Did the Researchers Do and Find?:**

The research was carried out in Wandsworth, an area of southwest London that is considered to be “ethnically diverse.” Over 4,200 people with diabetes are registered with general practitioners in this area. The researchers used the electronic records kept by these doctors and they focused on diabetes “treatment targets” set by the government, according to which the blood pressure and cholesterol levels of people with diabetes should be kept below defined levels. There is also a target level for glycated hemoglobin (HbA_1c_), which is a substance that can be used to measure the extent to which a patient's diabetes is under control. The researchers calculated the percentage of patients who were meeting these treatment targets. Overall, more patients met their treatment targets after the introduction of pay-for-performance management than were doing so before. All ethnic groups seemed to have benefited, but the black Caribbean group did not benefit as much as the other groups; the number of these patients who met the targets did improve, but the gap between them and patients with diabetes from other ethnic groups remained about the same.

**What Do These Findings Mean?:**

The researchers concluded that, while the introduction of pay-for-performance did seem to have been beneficial, it had not addressed disparities in the management and control of diabetes between ethnic groups. They say that, in all initiatives to improve the quality of health care, special efforts must be made to reduce such gaps. The UK's use of pay-for-performance in general practice is regarded internationally as a very bold step, but, as other countries are also considering moving in this direction, the lessons from the study will be relevant in many other parts of the world.

**Additional Information.:**

Please access these Web sites via the online version of this summary at http://dx.doi.org/10.1371/journal.pmed.0040191.

- Wikipedia has an entry on pay-for-performance in health care (note: Wikipedia is a free online encyclopedia that anyone can edit)
- Information about how the NHS works in England
- Diabetes UK is the largest organization in the UK working for people with diabetes and its website includes a useful Guide to Diabetes
- The London Health Observatory is one of nine health observatories set up by the NHS to monitor health and health care in England. There is a page devoted to “ethnic health intelligence”
- Introductory information about diabetes as a medical condition may be found on the MedlinePlus website; there are several MedlinePlus pages on diabetes as well

## Introduction

Internationally, there has been a drive to reduce persistent health disparities among minority ethnic populations, particularly in the United Kingdom and the United States [1]. In the UK, the government has recognised the importance of ensuring that new health policies are applied to all sectors of the population, including minority ethnic communities [2]. This consistent application is essential for policies directed at tackling the escalating diabetes epidemic in developed countries such as the UK and US, where diabetes is much more common in minority ethnic groups than in the general population [3,4]. Furthermore, these communities are likely to experience a disproportionate share of the future projected growth in the number of people with diabetes [3,4]. This differential burden in prevalence is exacerbated by higher complication rates and a worse morbidity and mortality profile amongst minority ethnic groups when compared with white patients [5,6]. Because of these disparities, diabetes contributes substantially to the variations in all-cause mortality between ethnic groups [7].

Quality improvement strategies have been shown to improve diabetes management and control [8,9]. A recent systematic review found that case management, patient reminders, patient and clinician education, audits, and electronic registers produced small to modest improvements in glycaemic control in individuals with type 2 diabetes [9]. However, these strategies appear to be less successful in addressing variations in care across socioeconomic and ethnic groups [10,11]. In addition, available evidence suggests that the publication of several major clinical trials on optimum diabetes management during the 1990s and subsequent development of national diabetes guidelines have not reduced variations in hypertension and glycaemic control between ethnic groups [12,13].

The use of pay-for-performance incentive schemes as a quality improvement tool is increasing, particularly in the US and UK [14,15]. Such schemes aim to improve the quality of health care for all patients so that it meets established standards, and therefore they provide an important opportunity to address disparities in chronic disease management between different ethnic and socioeconomic groups [16,17]. Few previous evaluations of pay-for-performance schemes have focused on chronic disease management. Furthermore, most studies have been carried out in the US and thus international studies are lacking [14]. A new contract for general practitioners, introduced in the UK in April 2004, represents the most radical shift towards pay-for-performance seen in any health-care system in the world [18]. This unique quality improvement initiative has been supported by £1.8 billion additional investment in primary care and provides an opportunity to examine the impact of pay-for-performance on addressing current disparities in health care.

In this paper, we examine diabetes management and outcomes using individual patient-level data in a multiethnic population before and after the introduction of a major performance incentive scheme. The findings are potentially important for other health-care systems, as pay-for-performance is seen as one method of improving the quality of primary care services to disadvantaged groups and thus of reducing health disparities.

## Methods

### Pay-for-Performance in UK Primary Care

Pay-for-performance was introduced in UK primary care as part of the new General Practitioner contract in April 2004 [18]. About one-fourth of general practice income is now derived through the achievement of quality targets in managing chronic diseases such as diabetes, stroke, and coronary heart disease through the Quality and Outcomes Framework. The Framework consists of 1,050 points, which cover clinical care, practice organisation, and patient experience.

Diabetes is one of ten disease areas within the clinical domain of the Quality and Outcomes Framework (although the number of indicators areas increased from ten to nineteen when the contract was revised in April 2006). Of the 99 points available for diabetes care, 50 are allocated for the achievement of treatment targets (blood pressure [BP] ≤ 145/85 mm Hg, 17 points; HbA_1c_ ≤ 7.4%, 16 points; HbA_1c_ ≤ 10%, 11 points; cholesterol ≤ 5 mmol/l or 193 mg/dl, 6 points) and the remainder to the recording of 14 process measures of care, including ascertainment of smoking status (3 points), annual measurement of body mass index (3 points), and retinopathy screening (5 points).

### Wandsworth Prospective Diabetes Study

In England, the provision of primary care services is the responsibility of primary care trusts. There are approximately 150 primary care trusts in England, each of which typically covers a population of 300,000 to 400,000 people. Within each primary care trust, primary care services are delivered by general practitioners working in National Health Service (NHS) general practices. Through the Wandsworth Prospective Diabetes Study (WPDS), the Wandsworth Primary Care Trust, located in southwest London, has established comprehensive primary care-based diabetes registers. Data for the present study were collected both before (June–October 2003) and after (November 2005–January 2006) the introduction of the new general practitioner contract in the UK in April 2004. Ethical approval for the study was granted by the Wandsworth Local Research Ethics Committee.

### Setting and Participants

The study area contained 36 general practices with a registered population of 243,519 patients. The median list size of practices was 6,349 patients and there was an even distribution of large, medium, and small practices in the study area (13 practices had more than 8,000 patients, 13 practices had between 3,000 and 8,000 patients, and ten practices had fewer than 3,000 patients).

The population of Wandsworth is younger than that of England as a whole, with 74% of people under age 45 y (compared with a national average of 60%). Approximately one in five Wandsworth residents (22%) belong to a nonwhite ethnic group. Of these, 4.9% are black Caribbean, 3.9% are black African, 2.9% are Indian, 2.1% are Pakistani, and 0.4% are Bangladeshi. Wandsworth has high levels of disparities in income relative to elsewhere in England [19].

### Identification of People with Diabetes

The methods we used to develop our disease register for diabetes in Wandsworth have been described previously [20]. In brief, we approached all practices in the study area to participate. All patients with type 1 and type 2 diabetes mellitus were identified from computerized general practice records in participating practices by searching for diagnoses of diabetes (C10) or diabetes care (66A) Read codes. Patients with repeat prescribing for diabetic medications, or with an HbA_1c_ greater than 7.4%, were also included in our sample. Patients under 18 y of age, or women with gestational diabetes or receiving treatment for polycystic ovarian syndrome rather than diabetes, were excluded. A unique patient identifier (NHS Number) was used to link patient records extracted in both collection periods.

### Study Variables

We examined prescribing levels and intermediate clinical outcome indicators for diabetes as they applied to our population in 2003 and 2005. Each indicator is based on clinical information recorded on the practice computer. Intermediate outcome indicators were included if they were recorded during the previous 15 mo. Patients self-identified their ethnic origin from closed categories based on the classifications that map to those used in the 2001 UK census [21], either at registration or during a consultation at the general practice. We assigned socioeconomic status to individual patients based on their postcode using the Index of Multiple Deprivation 2004 [19]. The Index of Multiple Deprivation is the most commonly used method of measuring neighbourhood socioeconomic status in the UK and is compiled from a range of sources, including the 2001 UK census, unemployment, and social security benefits records.

### Statistical Analyses

Our analyses took into account that we have repeated measurements for each patient, patients were clustered within practices, and ethnic minority groups varied in their age distributions. For each indicator, we described percentage achievement of quality indicators in each ethnic group. We used the McNemar test to examine overall differences in the frequency distributions of indicators between 2003 and 2005. To determine the association between ethnicity and achievement of quality indicators we used conditional logistic regression specifying for the clustering of patients within practices. We studied the association of change in achievement with ethnicity by conditioning the 2005 achievement on the 2003 achievement in a conditional logistic model. All standard errors are robust [22]. As we have measurements at two time points, our findings could be influenced by a regression to the mean effect [23]. To test for this effect we did a sensitivity analysis by repeating our analyses after stratifying the data according to the outcome for each indicator in the first time point. In doing this, we were looking for any substantive differences in results between the two strata. All analyses were undertaken using the Stata 9.1 program (Stata Corporation, http://www.stata.com).

## Results

We identified 4,284 adults (aged ≥ 18 y) with diabetes registered with the 32 participating practices in both 2003 and 2005. Included were 2,227 men and 2,057 women. The European age-standardised prevalence of diabetes in 2005 was 42.2 per 1,000 people in all age groups. Ethnicity was recorded in 95.1% of the sample (Table S1). Overall, the four practices that did not participate in the study accounted for less than 6% of the registered population in the study area. Nonparticipating practices were smaller (three of the four had fewer than 3,000 patients) and were located in more deprived areas than the participating practices. Our findings were substantially unchanged when we applied sensitivity analyses to test for the effect of measurement at two time points.

### Hyperglycaemia Management and Control

Significantly more patients (McNemar test, *p* = 0.005) achieved recommended levels for HbA_1c_ in 2005 (37.4%) than in 2003 (35.1%). These changes were uniform across ethnic groups, except in members of the black Caribbean group, who had significantly less improvement in glycaemic control than did the white British group after adjusting for age, gender, deprivation, and practice-level clustering (Table 1). Worse HbA_1c_ control in south Asian and black groups relative to the white British group persisted between 2003 and 2005.

**Table 1:**
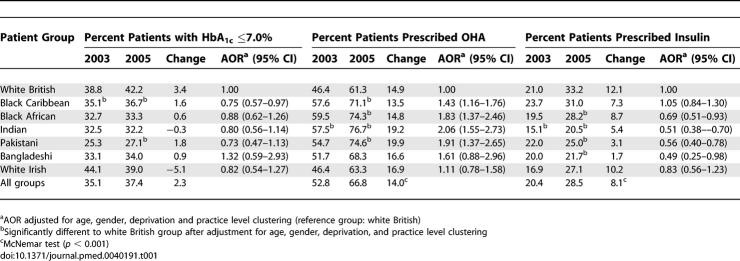
Ethnic Disparities in Glycaemic Management and Control

Significantly more patients (McNemar test, *p* < 0.001) were treated with insulin in 2005 (28.5%) than in 2003 (20.4%). However, increases in insulin prescribing were significantly lower within the black African and south Asian groups than in the white British group, resulting in a widening of the variation existing before the new contract. Significantly more patients (McNemar test, *p* < 0.001) were treated with oral hypoglycaemic agents (OHAs) in 2005 (66.8%) than in 2003 (52.8%). These increases were significantly larger in all black and south Asian patient groups relative to white British patients, resulting in widening of the existing variation between ethnic groups evident before the new contract was implemented.

### Hyperlipidemia Management and Control

Significantly more patients (McNemar test, *p* < 0.001) met the treatment target for total cholesterol in 2005 (70.4%) than in 2003 (57.5%). These changes were uniform across ethnic groups, with the exception of the Bangladeshi group, who had significantly greater improvement in cholesterol control relative to the white British group after adjusting for age, gender, deprivation, and practice-level clustering (Table 2). A worse lipid profile evident within the white British group in 2003 was not attenuated in 2005. Significantly more patients (McNemar test, *p* < 0.001) were treated with lipid-lowering agents in 2005 (59.7%) than in 2003 (37.8%). Lower prescribing of lipid-lowering agents evident in the black African group in 2003 was not attenuated in 2005.

**Table 2:**
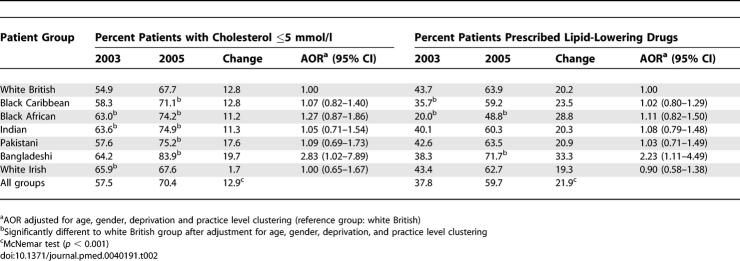
Ethnic Disparities in Cholesterol Management and Control

### Hypertension Management and Control

Significantly more patients (McNemar test, *p* < 0.001) met the treatment target for BP control in 2005 (42.3%) than in 2003 (31.4%). These changes were uniform across ethnic groups, with the exception of the black Caribbean group, who had significantly less improvement in BP control than did the white British group after adjusting for age, gender, deprivation, and practice-level clustering (Table 3). The worse BP profile evident in the black Caribbean group in 2003 was not attenuated in 2005. Significantly more patients (McNemar test, *p* < 0.001) were treated with an angiotensin-converting enzyme (ACE) inhibitor in 2005 (58.2%) than in 2003 (46.0%) and the increases were uniform across ethnic groups.

**Table 3:**
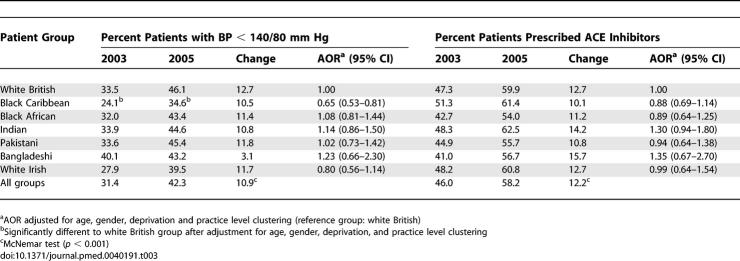
Ethnic Disparities in BP Management and Control

## Discussion

### Main Findings

The proportion of patients reaching treatment targets for HbA_1c_, BP, and total cholesterol increased significantly after the implementation of a new contract for general practitioners in the UK that linked pay to performance. The increases were broadly uniform across ethnic groups, except for the black Caribbean group, which had improvements in HbA_1c_ and BP control that were significantly lower than in the white British group. Variations in prescribing and achievement of treatment targets between ethnic groups evident in 2003 were not attenuated in 2005.

### Comparison with Previous Research

Few studies have examined the impact of pay-for-performance incentives on variations in chronic disease management and outcomes [14,15]. Complementary quality improvement strategies in diabetes management have shown small to modest improvements in glycaemic management in controlled trial settings [8]. However, the impact of such strategies in population settings appears mixed, with some evidence of overall improvement in care in countries such as the UK and Sweden [24,25], but more equivocal results in the US [26,27]. Most previous studies highlight persisting variations in diabetes management and outcomes between ethnic groups. For example, findings from the National Health and Nutrition Examination Survey (NHANES) in the US suggest that poorer glycaemic control evident in black and Mexican American participants relative to whites in the 1988–1994 survey had not been attenuated in 1999–2000, despite publication of national clinical guidance and other quality initiatives in the interim period [12]. Similarly, McElduff and colleagues [13] identified persistently higher HbA_1c_ levels amongst south Asians compared with Europeans attending primary and secondary care settings in Blackburn, northwest England, throughout a comparable time period (1995–2001).

### Strengths and Limitations

We are not able to demonstrate a definitive causal association between pay-for-performance incentives (introduced in April 2004) and the changes in diabetes management between 2003 and 2005. This is due to limitations of our study design; an observational study with two time points and no control group. Because the new contract for general practitioners in the UK was introduced nationally, evaluation of these incentives using a more rigorous study design, such as a randomised controlled trial, was not feasible. Recent observational studies indicate that the management of diabetes in primary care was improving in the UK before the introduction of the contract. However, the magnitudes of improvement seen before the contract was introduced are considerably lower than those reported here. For example, Campbell et al. [24] examined trends in diabetes management in 42 volunteer general practices in six geographical areas in England between 1998 and 2003. They reported that the percentage of people with diabetes meeting a BP target of 140/85 mm Hg (a less stringent target than the 140/80 mm Hg we used) increased from 21.8% to 35.8% (an annual improvement of 2.3%) over this period. In a separate study of 74 general practices in England and Wales, de Lusignan et al [28] found that the percentage of people with diabetes with a BP < 140/80 mm Hg increased from 13.6% in 1994 to 21.8% in 2001, an annual improvement of 1.2% per year. This result contrasts with an annual improvement seen in our study of 5.5% (from 31.4% to 42.3%). Similarly, improvements in HbA_1c_ control were more marked in our study population than those observed in these previous studies. Improvements seen after the introduction of pay-for-performance incentives are even more impressive because early improvements in quality tend to be the easiest to achieve.

To our knowledge this is the first population-based, longitudinal study in the UK to examine changes in diabetes management in a multiethnic population using individual patient level data. Most previous research examining quality of diabetes care has either not been population based [13,29], or compared outcomes using two or more cross-sectional surveys containing groups of patients that may differ systematically [25,26]. Use of routine clinical data means that there may have been some variability in the completeness and accuracy of the information collected [30]. We were also unable to adjust for certain patient factors, such as duration of diabetes, presence or severity of complications, and diabetes type, which may have been confounders in the relationship between ethnicity and diabetes management. We were able to adjust for differences in deprivation between ethnic groups and clustering of patients in primary care practices. However, adjusted findings were not substantially different from those based on unadjusted analyses (unpublished data). Assigning patients an area-based deprivation score may have masked actual differences in affluence and educational attainment both within and between ethnic groups.

We had sufficient numbers within each ethnic group to avoid the known limitations of combining individuals from heterogeneous populations into a single ethnic category, such as “south Asians” [31]. However, we acknowledge that some of the comparisons made may not have reached statistical significance due to small numbers in certain groups, for example, Bangladeshis. Our findings may represent a more complete picture of diabetes management than that derived from national contract data, which determine individual practice income and may exclude a considerable proportion of patients who have been exception reported by practices for poor treatment compliance [32]. People with diabetes were identified from computerised records using algorithms based upon diagnostic and diabetes care codes. We have previously shown that computer searches based on diagnostic Read codes for diabetes alone have a low sensitivity, as they may miss up to one-third of cases [20]. We used a more comprehensive search strategy to compensate for this under-recording of diabetes. All but four general practices within the study area participated in our survey. Hence our findings provide a comprehensive and typical picture of the care provided in this diverse, inner city location.

### Policy Implications

Our findings suggest that the implementation of a new contract for primary care physicians, which is one part of a comprehensive policy drive to improve the quality of chronic disease management within the National Health Service since 1997 [33], may have failed to address known disparities in diabetes management and outcomes between ethnic groups. Should we thus conclude that the principal mechanisms for attenuating these differential outcomes lie outside the reach of health-care systems [11]? The definitive answer to this question lies beyond the scope of this study, but it is worth reflecting on the following points. First, we found evidence of differential management of hyperglycaemia across ethnic groups, with lower relative increases in insulin prescribing in minority ethnic patients when compared with white patients. This pattern may not be solely due to provider factors, given that patients from minority ethnic groups identify numerous barriers to quality diabetes care [34,35]. Whilst this finding was coupled with higher levels of OHA prescribing amongst minority ethnic groups when compared with white patients, we were unable to examine adherence to medications or self-monitoring behaviour, which may systematically differ between the groups we studied [35,36]. Second, the current configuration of incentives within the new contract may provide insufficient rewards for practices working to achieve the key treatment targets for diabetes in ethnically diverse areas. At present, only one-fourth of practice income is derived through the quality and outcomes framework, of which approximately 50% is dependent on the achievement of treatment targets. Third, our analysis is based on data extracted 18 mo after the implementation of the new contract. This may be too soon to assess the full impact of pay-for-performance incentives on disparities in diabetes outcomes. Finally, the UK Prospective Diabetes Study (UKPDS) found similar glycaemic control amongst white, black, and south Asian patients at baseline and 9 y follow-up [37]. However, clinical trials include only a minority of patients with diabetes and may not be fully representative. Our findings highlight the importance of routinely recording ethnicity in health-care information systems and the ongoing need for local equity audits examining disparities in health-care access.

### Conclusions

There remains considerable scope to improve the management of diabetes in minority ethnic communities if the patterns of care and outcomes identified in this study apply elsewhere. Although diabetes management improved in all ethnic groups after the introduction of pay-for-performance incentives in UK primary care, disparities in prescribing and intermediate clinical outcomes persisted. Hence, the main lesson from this study for health-care systems in other countries is that pay-for-performance by itself may not be sufficient to address ethnic disparities in the quality of care. Consequently, future quality improvement initiatives must place greater emphasis on minority communities. This effort may help prevent continued disparities in mortality from cardiovascular disease and the other major complications of diabetes. However, the optimal methods for addressing ethnic disparities in health remain unclear, and this is an area that would benefit from further high-quality interventional and observational studies.

## Supporting Information

Table S1 Characteristics of Patients (34 KB XLS)

## Abbreviations

ACE: angiotensin-converting enzyme
AOR: adjusted odds ratio
BP: blood pressure
95% CI: 95% confidence interval
OHA: oral hyperglycaemic agent
